# Prevalence and factors associated with one-year mortality of infectious diseases among elderly emergency department patients in a middle-income country

**DOI:** 10.1186/s12879-019-4301-z

**Published:** 2019-07-25

**Authors:** Maythita Ittisanyakorn, Sukkhum Ruchichanantakul, Alissara Vanichkulbodee, Jiraporn Sri-on

**Affiliations:** 1 The Department of Emergency Medicine, Vajira Hospital, Navamindradhiraj University, Bangkok, Thailand; 2 The Department of Emergency Medicine, Chaophya Abhaibhubejhr Hospital, Bangkok, Thailand

**Keywords:** Infectious diseases, Emergency department, Elderly patients, Mortality rate

## Abstract

**Background:**

This study aimed to determine the prevalence of infectious diseases and risk factors for one-year mortality in elderly emergency department (ED) patients.

**Methods:**

A retrospective cohort study of patients aged 65 and over who visited the ED of one urban teaching hospital in Bangkok, Thailand and who were diagnosed with infectious diseases between 1 January 2016 and 30 June 2016.

**Results:**

There were 463 elderly patients who visited ED with infectious diseases, accounting for 14.5% (463/3,196) of all elderly patients’ visits. The most common diseases diagnosed by emergency physicians (EPs) were pneumonia [151 (32.6%) patients] followed by pyelonephritis [107 (23.1%) patients] and intestinal infection [53 (11.4%) patients]. Moreover, 286 (61.8%) patients were admitted during the study period. The in-hospital mortality rate was 22.7%. 181 (39.1%) patients died within 1 year. Our multivariate analysis showed that age 85 years and older [odds ratio (OR) = 1.89; 95% confidence interval (CI): 1.36–2.63], Charlson Co-morbidity Index score ≥ 5 (OR = 3.51; 95% CI2.14–5.77), lactate ≥4 mmol/l (OR = 2.66;95% CI 1.32–5.38), quick Sequential Organ Failure Assessment (qSOFA) score ≥ 2 (OR = 5.46; 95% CI 2.94–10.12), and platelet count < 100,000 cells/mm^3^ (OR = 3.19; 95% CI 1.15–8.83) were associated with 1-year mortality.

**Conclusions:**

In one middle-income country, infectious diseases account for 14.5% of elderly ED patients. Almost two-thirds of patients presenting to ED with infection are admitted to hospital. One-third of elderly ED patients with infection died within 1 year. Age ≥ 85 years, Charlson Co-morbidity Index score ≥ 5, lactate ≥4 mmol/l, qSOFA score ≥ 2, and platelet count < 100,000 cells/mm^3^ predicted 1-year mortality rate.

## Background

Infectious diseases (IDs) are some of the most common causes of death worldwide [1, 2]. Overall the trend of infectious diseases in developed countries is a decline, such as in the US; infectious diseases accounted for 797:100,000 population in 1900 and declined to 97:100,000 population in 1996 [3, 4]. In contrast, when focusing on elderly populations in 1990–2002, the rate of hospital admission for IDs increased to 13% [5]. One epidemiological study of elderly emergency department (ED) patients with IDs showed a resulting hospital admission rate of 57.2% [6]. Likewise, an Israeli study from 2011 found an increased rate of hospital admission among older patients to 14.2% and the most common disease was lower respiratory tract infection, accounting for 41%. A study in the Netherlands found the oldest-old populations (age ≥ 85 years) who were independent in activities of daily living (ADL) beacame less able in ADLs with a diagnosis of infectious disease [7]. One study in Canada which assessed the temporal trend of *salmonella* infection found the incidence of infection in seniors could increase by 16% by 2018 [8].

Accelerated population aging is now well-established in many middle-income countries leading to an increased number of older adults. Thailand, a middle-income country, has an aging population will account for one-third of its total population in 2040 [9–11]. IDs were the second most common causes of death for Thai people. The mortality rate was 41:100,000 population in 2009 [12]. Lower respiratory tract infection was the most common infection [12]. Most Thai research on IDs has focused on specific diseases and in-hospital admission may limit the importance of follow-up ED visits [12–14].

To address the gap, we conducted a study to determine the prevalence of infectious diseases and risk factors for one-year mortality in elderly ED patients in one middle-income country.

## Methods

### Design and setting

This was a retrospective cohort study. We reviewed data of all patients aged 65 and older who had a diagnosis related to infectious disease between 1 January 2016 and 30 June 2016 and received treatment at one ED of a university hospital in Bangkok, Thailand. Our hospital has approximately 50,000 ED visits per year and 18% of them are aged over 65 years. Patients with infectious diseases were identified initially by searching the hospital’s electronic database using International Classification of Diseases 10th (ICD-10). The ICD-10 code were defined in supplement 1.

Exclusion criteria were patients with unspecified diagnoses such as fever unspecified, diarrhea unspecified, Systemic Inflammatory Response Syndrome (SIRS), patients who transferred to other hospitals, patients triaged in ED as a non-urgent.

### Definitions

Polypharmacy was defined as the number of patients’ medications ≥5.

Sepsis fast track at this hospital was defined as patients who had at least 2 from 3 points of Systemic Inflammatory Response Syndrome (SIRS) criteria at triage; nurses then activated fast track by notifying EPs. SIRS at triage was defined as 1. body temperature < 36 °C or > 38 °C; 2. heart rate > 90 beats/min; and 3. respiratory rate (RR) > 20/min.

Quick Sequential Organ Failure Assessment (qSOFA) was defined as 1. systolic blood pressure (SBP) ≤ 100 mmHg; 2. respiratory rate ≥ 22/min; and 3. glasgow coma scale (GCS) < 15.

### Data collection process

The data collection was done by a third-year emergency resident, medical students in their sixth year, and a registered nurse who had three years’ practicing experience in ED. Data were extracted from electronic medical records (EMR), which included ED diagnosis, laboratory information system, and ICD-10 codes. For in-hospital patients, we extracted diagnostic data from summaries of the notes of resident doctors who were in charge of each ward. Our hospital has a policy that attending physicians recheck diagnoses.

### Research assistants training process

Medical students and the registered nurse [research assistants (RAs)] were trained to collect data under supervision of the principle investigator (PI). This included three hours’ training for data collection and identifying medical terms. RAs met the principle investigator once a month to clarify terms and data that were not clear. Furthermore, they could contact PI directly if they had problems with the terms or were unsure about data abstraction. The PI randomly selected 5% of medical records to test for interrater reliability between RAs for the subjective variables such as ED diagnosis. Kappa statistic was 0.84.

The collected data consisted of age, gender, educations level, underlying diseases, number of medications, type of medications, Charlson co-morbidity index [15], activities of daily living (ADL) [16], modified Canadian triage level [17], activated sepsis fast track, quick Sequential Organ Failure Assessment (qSOFA) [18], vital signs, hemoculture, lactate, other specimen cultures such as sputum, urine, time to receive antibiotic, hospital admission rate, and in-hospital mortality rate. One-year mortality rate was determine by using database from Thailand Office of Central Civil Registration and hospital database, where available. All Thai people are registered to the system after their birth and given an identification number; after death the government also records this in the system.

Patients’ informed consent was waived by the ethics committee of our hospital, since approval is not considered necessary for analyzing anonymous data for quality management. This study was approved by the hospital’s institutional review board.

### Statistical analysis

Quantitative values such as age, charlson comorbidity index score were presented using mean and standard deviation (SD) or median and interquartile range (IQR) where appropriate. The relationship between factors was determined by using student’s t-test if the data were normally distributed or Mann Whitney u test if the data were non-normally distributed. The calculation was statistically significant when *p*-value was less than 0.05. Qualitative values such as gender, hospital admission rate, and mortality rate were presented using percentages. Chi-square was used to test a relationship between factors, with *p*-value less than 0.05 being statistically significant. Multiple logistic regression analysis was used to determine risk factors associated with one-year mortality rate. The variables with a *p*-value < 0.1 from univariate analysis were chosen for the final model and analyzed using backward selection methods. Hosmer-Lemeshow goodness of fit was used to determine the fit of the model. All statistical calculations found in this study were calculated by STATA software version 13.0.

## Results

**Fig. 1:**
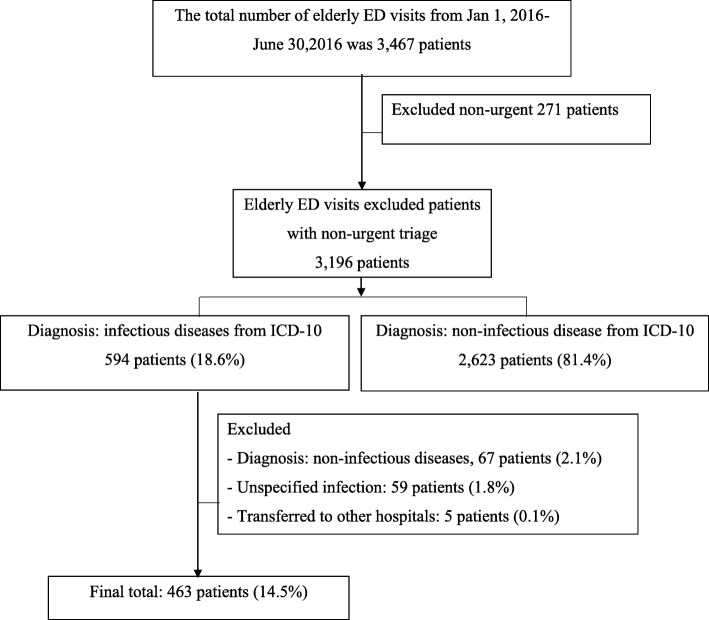
Enrollment of patients to study

There were 3,467 elderly patients who visited ED between 1st January 2016 and 30th June 2016. 3,196 patients were triaged as urgent, emergency and resuscitation. There were 594 patients who had diagnosis of infectious diseases following ICD-10. This study excluded 67 patients with non-infectious diseases, 59 patients with unspecified infection and 5 patients were transferred to other hospitals. Finally, 463 elderly patients were diagnosed with infectious diseases, accounting for 14.5% (463/3,196) of all elderly ED patients’ visits (Fig. 1).

Median age was 78 years (IQR 72–84). Most of the elderly patients arrived by family car [341 (73.7%) patients]. 146 (31.5%) patients had engagement in sepsis fast track. Median charlson co-morbidity index was 5 (IQR 4–6) (Table 1).

**Table 1:** Baseline characteristics, physical examination and investigation

Variable (*n* = 463)	N	(%)
Gender, male	204	(44.1)
Age, median (IQR) years	78	72–84
Patient’s insurance		
30 Baht healthcare scheme^a^	226	(48.8)
Social security	9	(1.9)
Government employee	163	(35.2)
Self-pay	65	(14.0)
Mode of arrival		
Family car	341	(73.7)
Ambulance	68	(14.7)
Others	54	(11.6)
Triage level		
Urgent	253	(54.6)
Emergency	181	(37.8)
Resuscitation	36	(7.8)
Charlson comorbidity index, median (IQR)	5	4–6
Polypharmacy (≥ 5 medications)	175	(37.8)
Sepsis fast track	146	(31.5)
Temperature (°C), *n* = 451		
36–38 °C	297	(65.9)
< 36 or > 38 °C	154	(34.1)
Heart rate (beat/min), *n* = 462		
> 90 /min	252	(54.3)
≤ 90/min	211	(45.7)
Respiratory rate (/min), *n* = 455		
> 20/min	291	(64.0)
≤ 20/min	164	(36.0)
Systolic blood pressure (mmHg), *n* = 462		
≤ 100	53	(11.5)
> 100	409	(88.5)
Oxygen saturation, *n* = 406		
< 90%	54	(13.3)
90–93%	51	(12.6)
≥ 94%	301	(74.1)
Systemic inflammatory response syndrome [SIRS (points)]		
0–1	169	(36.5)
2	132	(28.5)
3	107	(23.1)
4	55	(11.9)
Quick sequential organ failure assessment [qSOFA (points)]		
0–1	373	(80.5)
2	75	(16.2)
3	15	(3.2)
Hemoglobin (mg/dl), *n* = 450		
< 7	22	(4.9)
Platelet (cells/cm^3^), *n* = 449		
< 100,000	28	(6.2)
Creatinine (mg/dl), *n* = 444		
≥ 2	78	(17.6)
Creatinine clearance [CrCl (ml/min)], *n* = 445		
> 50	254	(57.1)
10–50	173	(38.9)
< 10	18	(4.0)
Blood glucose (mg/dl), *n* = 345		
≥ 180	97	(28.1)
Sodium (mmol/dl), *n* = 441		
< 135	141	(32.0)
135–145	283	(64.1)
> 145	17	(3.9)
Total bilirubin (mg/dl), *n* = 225		
≥ 2	26	(11.6)
International normalized ratio (INR), *n* = 240		
> 1.5	25	(10.4)
Lactate (mmol/l), *n* = 361		
≥ 2	220	(60.9)
≥ 4	59	(16.3)

^a^30 Baht healthcare scheme: Thailand has universal coverage healthcare which covers all medical expenses for the Thai populations

The most common diseases diagnosed by emergency physicians (EPs) were: pneumonia [151 (32.6%) patients]; followed by pyelonephritis [107 (23.1%) patients]; and intestinal infection [53 (11.4%) patients]. Pneumonia [103 (36.0%) patients] was the most common cause for hospital admission, followed by pyelonephritis [63 (23.4%) patients] and intestinal infection [29 (10.1%) patients] (Table 2).

**Table 2:** Ten most commonly diagnosed infectious diseases in elderly ED patients and in-hospital diagnosis (n = 463 patients)

Diagnosis	Total		Age (years)					
			65–74		75–84		> 85	
	N	%	N	%	N	%	N	%
ED diagnosis								
1. Pneumonia	151	(32.6)	48	(10.4)	69	(14.9)	34	(7.3)
2. Pyelonephritis	107	(23.1)	38	(8.2)	45	(9.7)	24	(5.2)
3. Intestinal infection	53	(11.4)	29	(6.3)	16	(3.5)	8	(1.7)
4. Skin and soft tissue infection	41	(8.9)	13	(2.8)	14	(3.0)	14	(3.0)
5. Other lower respiratory tract infection	38	(8.2)	9	(1.9)	18	(3.9)	11	(2.4)
6. Sepsis	19	(4.1)	10	(2.2)	8	(1.7)	1	(0.2)
7. Cholecystitis	9	(1.9)	4	(0.9)	4	(0.9)	1	(0.2)
8. Lower urinary tract infection	9	(1.9)	3	(0.6)	3	(0.6)	3	(0.6)
9. Complication from medicine or surgery	6	(1.3)	2	(0.4)	3	(0.6)	1	(0.2)
10. Pulmonary tuberculosis	5	(1.1)	1	(0.2)	3	(0.6)	1	(0.2)
Hospital admission diagnosis								
1. Pneumonia	103	(36.0)	24	(8.4)	49	(17.1)	30	(10.5)
2. Pyelonephritis	67	(23.4)	21	(7.3)	30	(10.5)	16	(5.6)
3. Intestinal infection	29	(10.1)	14	(4.9)	9	(3.1)	6	(2.1)
4. Other lower respiratory tract infection	22	(7.7)	4	(1.4)	11	(3.8)	7	(2.4)
5. Skin and soft tissue infection	19	(6.6)	8	(2.8)	7	(2.4)	4	(1.4)
6. Sepsis	14	(4.9)	7	(2.4)	6	(2.1)	1	(0.3)
7. Cholecystitis	8	(2.8)	3	(1.0)	4	(1.4)	1	(0.3)
8. Complication from medicine or surgery	5	(1.7)	0	(0.0)	4	(1.4)	1	(0.3)
9. Pulmonary tuberculosis	3	(1.0)	1	(0.3)	1	(0.3)	1	(0.3)
10. Appendicitis	3	(1.0)	2	(0.7)	0	(0.0)	1	(0.3)

82/329 (20.9%) patients had a positive of hemoculture. 128/136 (94.1%) had a positive sputum culture. Urine cultures were positive in 162/251 (64.5%) patients, while pus cultures were positive in 9/9 (100%) patients. Influenza screening was positive in 5/30 (16.7%) patients.

Two-hundred and eighty-six (61.8%) patients were admitted during the study period. Median hospital length of stay was 8 days (IQR 5–15). The in-hospital mortality rate was 65 (22.7%) patients. 181(39.1%) patients died within 1 year (Table 3).

**Table 3:** Outcomes of infectious elderly ED patients

Variables	N (%)
ED disposition	
Home	95 (20.5)
ED observation	65 (14)
Other hospitals	13 (2.8)
Hospital admission	286 (61.8)
Death	1 (0.2)
Other	3 (0.6)
Hospital admission, *n* = 286 patients	
Hospital length of stay, median (IQR)	8 (5–15)
Hospital disposition, *n* = 286	
Home	219 (76.6)
Refer to other hospital	2 (0.7)
Death	65 (22.7)
Mortality rate at 1 year	
Death	181 (39.1)
Causes of death	
Pneumonia	46 (26.4)
Cancer	26 (14.9)
Urinary tract infection	6 (3.4)
Sepsis	20 (11.4)
Unknown	76 (43.7)

Our multivariate analysis showed that age 85 years and older [odds ratio (OR) = 1.89; 95% confidence interval (CI): 1.36–2.63, *p*-value < 0.001], charlson co-morbidity index score ≥ 5 (OR = 3.51; 95% CI2.14–5.77, *p*-value < 0.001), lactate ≥4 mmol/l (OR = 2.66;95%CI 1.32–5.38, *p*-value < 0.001), quick Sequential Organ Failure Assessment (qSOFA) score ≥ 2 (OR = 5.46; 95% CI 2.94–10.12, *p*-value =0.025), and platelet count < 100,000 cells/mm^3^ (OR = 3.19; 95% CI 1.15–8.83, *p* value < 0.001) were associated with 1-year mortality (Table 4).

**Table 4:** Factors associated with one-year mortality rate (*N* = 370)

Factors	Death at one year						Odds_adj_	
	Yes		No		Odds 95% CI (crude)			95% CI
	N	%	N	%				
Gender, male	91	(44.6)	113	(55.4)	1.50	1.03–2.18	–	–
Age ≥ 85 years	56	(54.4)	47	(45.6)	2.24	1.44–3.49	1.89	1.36–2.63
Charlson co-morbidity index ≥5	142	(52.4)	129	(47.6)	4.32	2.82–6.60	3.51	2.14–5.77
Polypharmacy	85	(39.1)	135	(61.4)	0.95	0.72–1.65	–	–
Received antibiotic > 3 h	33	(35.1)	61	(64.9)	0.68	0.42–1.11	–	–
Body temperature < 36 °C or > 38 °C	56	(36.4)	98	(63.6)	0.83	0.56–1.24	–	–
Heart rate > 90 beats/min	103	(40.9)	149	(59.1)	1.17	0.81–1.72	–	–
Respiratory rate > 20 /min	135	(46.4)	156	(53.6)	2.51	1.65–3.82	–	–
Systolic blood pressure ≤ 100 mmHg	35	(64.8)	19	(35.2)	3.50	1.90–6.40	–	–
Oxygen saturation < 90%	35	(64.8)	19	(35.2)	2.99	1.64–5.45	–	–
SIRS ≥2 points	124	(42.2)	170	(57.8)	1.43	0.97–2.12	–	–
qSOFA ≥2 points	69	(75.8)	22	(24.2)	7.28	4.29–12.35	5.46	2.94–10.12
Hemoglobin < 7 mg/dl	15	(68.2)	7	(31.8)	3.38	1.35–8.47	–	–
Sodium (Na) < 135 mmol/dl	67	(47.5)	74	(52.5)	1.51	1.01–2.28		
Platelet < 100,000 cells/cm^3^	21	(75.0)	7	(25.0)	4.89	2.03–11.77	3.19	1.15–8.84
Creatinine ≥2 mg/dl	43	(55.1)	35	(44.9)	2.03	1.24–3.33	–	–
Blood sugar > 180 mg/dl	40	(41.3)	57	(58.7)	0.87	0.54–1.39	–	–
Bilirubin ≥2 mg/dl	16	(61.5)	10	(38.5)	1.16	0.70–3.73	–	–
INR > 1.5	14	(56.0)	11	(44.0)	1.17	0.51–2.69	–	–
Lactate ≥4 mmol/l	41	(69.5)	18	(30.5)	3.26	1.79–5.95	2.66	1.32–5.38

## Discussion

The prevalence of infectious diseases among elderly ED patients in one middle-income country was 14.5%. Three most common infections were pneumonia, pyelonephritis and intestinal infection. This finding was similar to that of Goto T, et al., who studied infectious disease–related ED visits of elderly adults in the United States, 2011–2012 [6]. They found the prevalence of infectious diseases in elderly ED patients was 13.5% and the two most common infections were lower respiratory tract infection (26.2%) and urinary tract infection (25.3%). In contrast, the rate of intestinal infection in our study was higher than Goto’s study. The explanation may be due to the tropical climate that grows more organisms, such as *vibrio cholerae* [19]. The prevalence of elderly ED infection was less than a cohort study from The Netherlands, which found a 17% rate of infectious disease in patients aged equal to or more than 70 years [20].

Hospital admission rate in this study accounted for 61.8%, similar to Goto T, et al. (57.1%). On the other hand, the top three common causes of infection among admitted patients in this study were pneumonia (36%), pyelonephritis (23%), and intestinal infection (10%), whereas Goto’s study found sepsis (32%), lower respiratory tract infection (28%), and urinary tract infection (17%) were the top three causes of infection among admitted patients. Despite the utilization of the sepsis fast track system, 22% of admission patients still died in this study, exactly comparable with Rebelo M, et al., who studied in-hospital mortality in elderly patients with bacteremia admitted to an internal medicine ward in Portugal and also found a rate of 22% [21]. This contrasts with Goto T, et al. whose study found only 4% died in-hospital. These findings may be a reflection on healthcare systems with the culture and environment differ.

Thirty-nine percent of infectious elderly ED patients died within one year.

Age ≥ 85 were associated with one-year mortality rate. IDs among elderly patients are different from younger patients because of the immune response that reduces complement activity, decreases Naïve T-cells, as well as anatomic and physiological changes with aging such as decreased acid-base in gastric secretions, decreased estrogen in menopause, increased risk of urinary tract infection, and polypharmacy. Multiple comorbidities increase older adults’ susceptibility to IDs [22–24]. Charlson co-morbidity index > 5 predicted one-year mortality rate in our study, which was comparable with the results Murray SB, et al. [25] who found charlson co-morbidity index > 5 had a 40% one-year mortality rate. Platelet count less than 100,000 cells/cm^3^ also predicted one-year mortality rate, as noted in studies by Vincent JL, et al. [18] and Singer M, et al. [23]. qSOFA ≥2 points was associated with one-year mortality rate in this study, which was comparable with Singer M, et al. whose study found qSOFA predicted mortality in Sepsis-3[26]. Lactate concentration ≥ 4 mmol/l was associated with increased 1-year mortality rate, which was comparable to a study by Audren et al., which found lactate concentrations > 4 mmol/L had a specificity of 96% in predicting mortality in hospitalized non-hypotensive patients [27]. Other studies found higher serum lactate levels were associated with higher mortality rate [28–30]. Clinically, hyperlactatemia (≥ 4 mmol/l) can be considered a warning signal for organ dysfunction and a guide for medical intervention among elderly patients.

Although our hospital has a sepsis fast track, following the sepsis-3 recommendations, still one-fifth of older adults died within 1 year. Sepsis guidelines for elderly ED patients that focus on and oldest-old population with charlson co-morbidity index > 5, lactate concentration ≥ 4 mmol/l and qSOFA ≥2 points may be beneficial.

### Limitations

Due to the retrospective nature of this study, we could not know some information such as patients taking other medications besides those on the hospital record form and frailty informations. Our hospital did not performed a comprehensive geriatric assessment as a part of treatment on that time, the results may not generalized. We could not evaluate all causes of death as some of the data came from Thailand Office of Central Civil Registration, which records only the date of death. This study was a single-center study, the results may not be generalized. In multiple logistic regression analysis, we did not impute missing data because it may have widened CI if not missing completely at random (MCAR).

## Conclusion

In one middle-income country, infectious diseases accounted for 14.5% in elderly ED patients. Pneumonia was the most common infection. Two thirds of these patients were admitted to hospital. One third of elderly ED patients died within 1 year. Age ≥ 85 years, charlson co-morbidity Index score ≥ 5, lactate concentration ≥ 4 mmol/l, qSOFA score ≥ 2, and platelet count < 100,000 cells/mm^3^ predicted 1-year mortality rate. Future research should focus on interventions to reduce mortality from infectious diseases in elderly ED patients.

## Data Availability

Data can be obtained from the corresponding author upon reasonable request.

## Appendix 1

ICD-10 for inclusion; code A00-A09, A15-A19, A20-A28, A30-A99, B00-B09, B15-B99, G00-G09, G73.4, G94.0, H03, H13, H19.2, H22.0, H32.0, H60, H75.0, H94.0, I00-I02, I68.1, I98.0–0.1, J00-J06, J09-J18, J20-J22, J85-J86, K35-K37, K67, K75.0, K77.0, K81, L00-L08, L30.3, M00-M03, M60.0, N08.0, N10-N12, N13.6, N16.0, N29.1, N39.0, N45, N70, N74, N77.0–0.1.

**Table:**

ICD-10 codes	Definitions
A00-A-09	Intestinal infectious diseases
A15-A19	Tuberculosis
A20-A28	Certain zoonotic and bacterial diseases
A30-A99	A30-A40 Other bacterial diseases
	A50-A64 Infections with a predominantly
	sexual mode of transmission A65-A69 Other spirochaetal diseases
	A70-A74 Other diseases caused by chlamydiae
	A75-A79 Rickettsioses
	A80-A89 Viral infections of the central nervous system
	A90-A99 Arthropod-borne viral fevers and viral haemorrhagic fevers
B00-B09	Viral infections characterized by skin and mucous membrane lesions
B15-B99	B15-B 99Viral hepatitis
	B20-B24 Human immunodeficiency virus [HIV] disease
	B25-B34 Other viral diseases
	B35-B49 Mycoses
	B50-B64 Protozoal diseases
	B65-B83 Helminthiases
	B85-B89 Pediculosis, acariasis and other infestations
	B90-B94 Sequelae of infectious and parasitic diseases
	B95-B98 Bacterial, viral and other infectious agents
	B99-B99 Other infectious diseases
G00-G09	Inflammatory diseases of the central nervous system
G73.4	Myopathy in infectious and parasitic diseases classified elsewhere
G94.0	Hydrocephalus in infectious and parasitic diseases classified elsewhere
H03	Disorders of eyelid, lacrimal system and orbit
H13	Disorders of conjunctiva in diseases classified elsewhere
H19.2	Keratitis and keratoconjunctivitis in other infectious and parasite diseases classified elsewhere
H22.0	Iridocyclitis
H32.0	Chorioretinal inflammation in infectious and parasitic diseases classified elsewhere
H60	Otitis media
H75.0	Mastoiditis in infectious and parasitic diseases classified elsewhere
H94.0	Acoustic neuritis in infectious and parasitic diseases classified elsewhere
I00-I02	Acute rheumatic fever
I68.1	Cerebral arteritis in infectious and parasite diseases classified elsewhere
I98.0–0.1	Cardiovascular syphilis
J00-J06	Acute upper respiratory infections
J09-J18	Influenza and pneumonia
J20-J22	Other acute lower respiratory infections
J85-J86	Suppurative and necrotic conditions of lower respiratory tract
K35-K37	Diseases of appendix
K67	Disorder of peritoneum in infections disease classified elsewhere
K 75.0	Other inflammatory liver diseases
K77.0	Liver disorders in infections and parasitic diseases classified elsewhere
K81	Cholecystitis
L00-L08	Infections of the skin and subcutaneous tissue
L30.3	Infective dermatitis
M00-M03	Infectious arthropathies
M60.0	Myositis
N08.0	Glomerular disorders in diseases classified elsewhere
N10-N12	Acute tubule-interstitial nephritis
N13.6	Pyonephrosis
N 16.0	Renal tubulo-interstitial disorders in infectious and parasitic diseases classified elsewhere
N29.1	Other disorders of kidney and ureter in infectious and parasitic diseases classified elsewhere
N39.0	Urinary tract infection unspecified
N45	Orchitis, epididymitis and epididymo-orchitis with abscess
N70	Salpingitis and oophoritis
N74	Female pelvic inflammatory disorders in diseases classified elsewhere
N 77.0–0.1	Ulceration of vulva in infectious and parasitic diseases classified elsewhere Vaginitis, vulvitis and vulvovaginitis in infectious and parasitic diseases classified elsewhere

## Appendix 2

**Table 5:** Outcomes of infectious elderly ED patients

Variables	N (%)
ED disposition	
Home	95 (20.5)
ED observation	65 (14)
Other hospitals	13(2.8)
Hospital admission	286 (61.8)
Death	1 (0.2)
Other	3(0.6)
Hospital admission, n = 286 patients	
Hospital length of stay, median (IQR)	8 (5–15)
Hospital disposition, n = 286	
Home	219 (76.6)
Refer to other hospital	2 (0.7)
Death	65 (22.7)
Mortality rate at 1 year	
Death	181 (39.1)
Causes of death	
Pneumonia	46 (26.4)
Cancer	26 (14.9)
Urinary tract infection	6 (3.4)
Sepsis	20 (11.4)
Unknown	76 (43.7)

## Abbreviations

ADL: Activity of daily living
CI: Confidence interval; OR: odds ratio
ED: Emergency department
EPs: Emergency physicians
ICD-10: International Classification of Diseases 10th
IDs: Infectious diseases
IQR: Interquartile range
PI: Principle investigator
qSOFA: quick Sequential Organ Failure Assessment
RAs: Research assistants
RR: Respiratory rate
SBP: Systolic blood pressure
SD: Standard deviation
SIRS: Systemic Inflammatory Response Syndrome
SpO_2_: Oxygen saturation

## References

[1] Armstrong GL, Conn LA, Pinner RW (1999). Trends in infectious disease mortality in the United States during the 20th century. JAMA.

[2] Saliba W, Fediai A, Edelstein H, Markel A, Raz R (2013). Trends in the burden of infectious disease hospitalizations among the elderly in the last decade. Eur J Intern Med.

[3] Christensen KL, Holman RC, Steiner CA, Sejvar JJ, Stoll BJ, Schonberger LB (2009). Infectious disease hospitalizations in the United States. Clin Infect Dis.

[4] Fry AM, Shay DK, Holman RC, Curns AT, Anderson LJ (2005). Trends in hospitalizations for pneumonia among persons aged 65 years or older in the United States, 1988-2002. JAMA.

[5] Curns AT, Holman RC, Sejvar JJ, Owings MF, Schonberger LB (2005). Infectious disease hospitalizations among older adults in the United States from 1990 through 2002. Arch Intern Med.

[6] Goto T, Yoshida K, Tsugawa Y, Camargo CA, Hasegawa K (2016). Infectious disease-related emergency department visits of elderly adults in the United States, 2011-2012. J Am Geriatr Soc.

[7] Caljouw MA, Kruijdenberg SJ, de Craen AJ, Cools HJ, den Elzen WP, Gussekloo J (2013). Clinically diagnosed infections predict disability in activities of daily living among the oldest-old in the general population: the Leiden 85-plus study. Age and Aging.

[8] Turgeon P, Ng V, Murray R, Nesbitt A (2018). Forecasting the incidence of salmonellosis in seniors in Canada: a trend analysis and the potential impact of the deographic shift. PLoS One.

[9] Ousvapoom MP, Suthiart MA, Aryukasam MT, Senpong MS. Statistical Thailand 2013: Bureau of Policy and Strategy. Ministry of Public Health. 2013; https://www.m-society.go.th/article_attach/11378/15693.pdf. Accessed 4 Apr 2018.

[10] Global health - Thailand. 2010. http://www.cdc.gov/globalhealth/countries/thailand/. Accessed 12 Jan 2016.

[11] Number of deaths by leading cause of death and sex, whole kingdom: 2007–2014. http://service.nso.go.th/nso/web/statseries/tables/00000_Whole_Kingdom/deaths-50-57.xls. Accessed 14 Jan 2019.

[12] Suchunya A, Margaret M, Jongkol L, Sonja JO, Kanitta B (2012). Infectious disease mortality rates, Thailand, 1958–2009. Emerg Infect Dis.

[13] Whistler T, Sapchookul P, McCormick DW, Sangwichian O, Jorakate P, Jatapai A (2018). Epidemiology and antimicrobial resistance of invasive non-typhoidol salmonellosis in rural Thailand from 2006-2014. PLoS Negl Trop Dis.

[14] Hantrakun V, Somayaji R, Teparrukkul P, Boonsri C, Rudd K, Day NPJ (2018). Clinical epidemiologyand outcomes of community acquired infection and sepsis among hospitalized patients in a resource limited setting in Northeast Thailand: a prospective observational study (Ubon-sepsis). PLoS One.

[15] Charlson ME, Pompei P, Ales KL, MacKenzie CR (1987). A new method of classifying prognostic comorbidity in longitudinal studies: development and validation. J Chronic Dis.

[16] Katz S, Ford AB, Moskowitz RW, Jackson BA, Jaffe MW (1963). Studies of illness in the aged: the index of ADL: a standardized measure of biological and psychosocial function. JAMA..

[17] Bullard MJ, Chan T, Brayman C, Warren D, Musgrave E, Unger B (2014). Revisions to the Canadian emergency department triage and acuity scale (CTAS) guidelines. CJEM.

[18] Vincent JL, de Mendonca A, Cantraine F, Mereno R, Takala J, Suter PM (1998). Use of the SOFA score to assess the incidence of organ dysfunction/failure in intensive care units: results of a multicenter, prospective study. Working group on “sepsis-related problems” of the European Society of Intensive Care Medicine. Crit Care Med.

[19] Tropical Diseases. http://www.astmh.org/education-resources/tropical-medicine-q-a/major-tropical-diseases. Accessed 15 Dec 2017.

[20] Schrijver EJ, Toppinga Q, de Vries OJ, Kramer MH, Nanayakkara PW (2013). An observational cohort study on geriatric patient profile in an emergency department in the Netherlands. Neth J Med.

[21] Rebelo M, Pereira B, Lima J, Decq-Mota J, Vieira JD, Costa JN (2011). Predictors of in-hospital mortality in elderly patients with bacteraemia admitted to an internal medicine ward. Int Arch Med.

[22] Ph K. Infection in the elderly. In: Jeffrey BH, Go J, Et M, S S, Ph K, A S, editors. Hazzaid’s geriatric medicine and gerontology. 6 ed. New york: McGraw-Hill; 2009.

[23] Birnbaumer DM, Marx JA, Hockberger RS, Walls RM (2013). The Elder Patient. Rosen's emergency medicine : concepts and clinical practice. 8 ed.

[24] Castle SC (2000). Clinical relevance of age-related immune dysfunction. Clin Infect Dis.

[25] Murray SB, Bates DW, Ngo L, Ufberg JW, Shapiro NI (2006). Charlson index is associated with one-year mortality in emergency department patients with suspected infection. Acad Emerg Med.

[26] Singer M, Deutschman CS, Shankar-Hari M, Annane D, Bauer M, Bellomo R (2016). The third international consensus definitions for sepsis and septic shock (sepsis-3). JAMA..

[27] Aduen J, Bernstein WK, Khastgir T, Miller J, Kerzner R, Bhatiani A (1994). The use and clinical importance of a substrate-specific electrode for rapid determination of blood lactate concentrations. JAMA..

[28] Singer AJ, Taylor M, Domingo A, Ghazipura S, Khorasonchi A, Thode HC (2014). Diagnostic characteristics of a clinical screening tool in combination with measuring bedside lactate level in emergency department patients with suspected sepsis. Acad Emerg Med.

[29] Mikkelsen ME, Miltiades AN, Gaieski DF, Goyal M, Fuchs BD, Shah CV (2009). Serum lactate is associated with mortality in severe sepsis independent of organ failure and shock. Crit Care Med.

[30] Rivers EP, Kruse JA, Jacobsen G, Shah K, Loomba M, Otero R (2007). The influence of early hemodynamic optimization on biomarker patterns of severe sepsis and septic shock. Crit Care Med.

